# Supplementary material for: Multi-Functional Silver Nanoparticles for High-Throughput Endospore Sensing
Source: Biosensors (Basel). 2022 Jan 25;12(2):68. doi: 10.3390/bios12020068 (PMC8869755; doi:10.3390/bios12020068)
Supplement: Supplementary file 1 [file biosensors-12-00068-s001.zip › biosensors-1559383-supplementary.pdf]

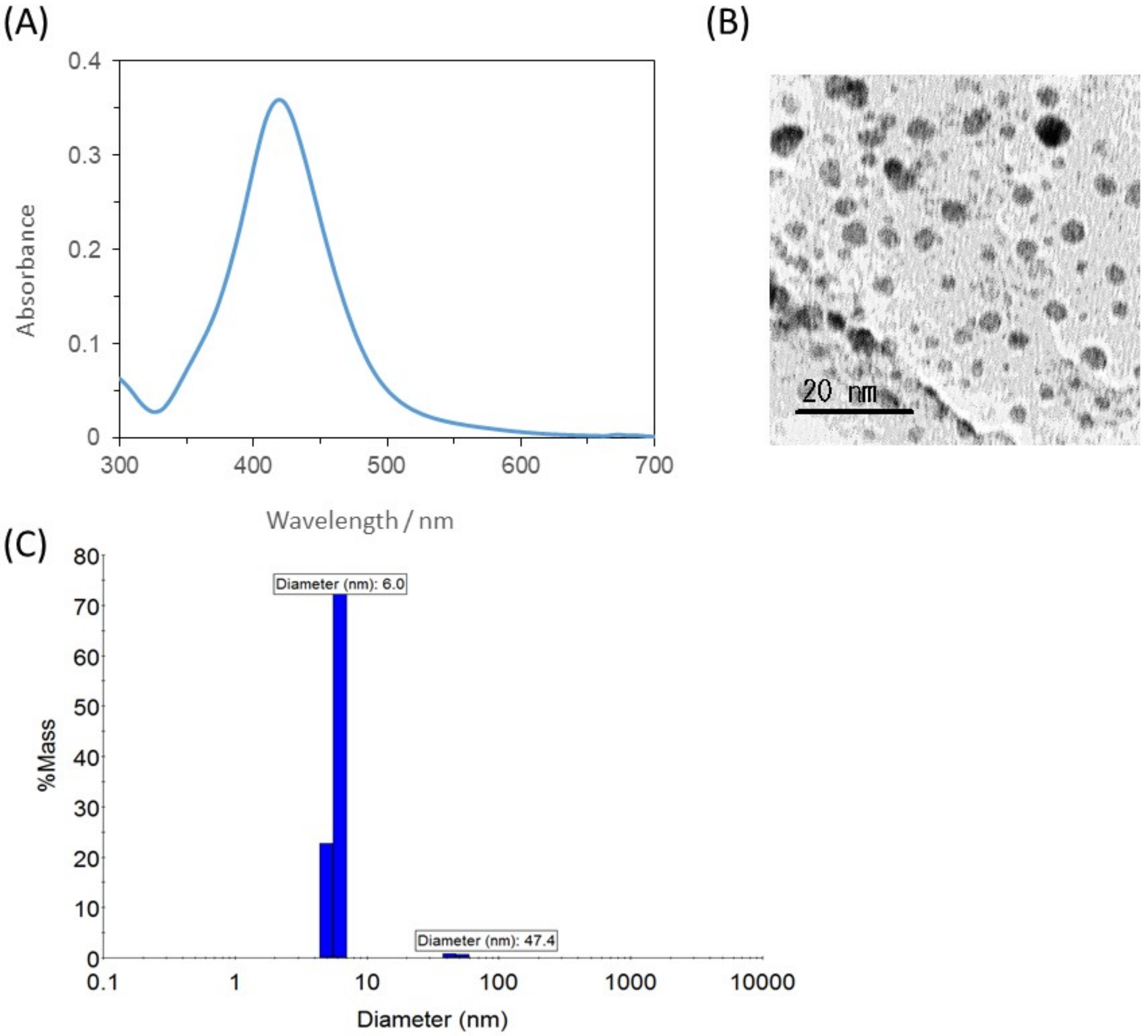

Figure S1 Characterization of Ag nanoparticles. (A) Absorbance spectrum (B) TEM image, and (C) DLS analysis

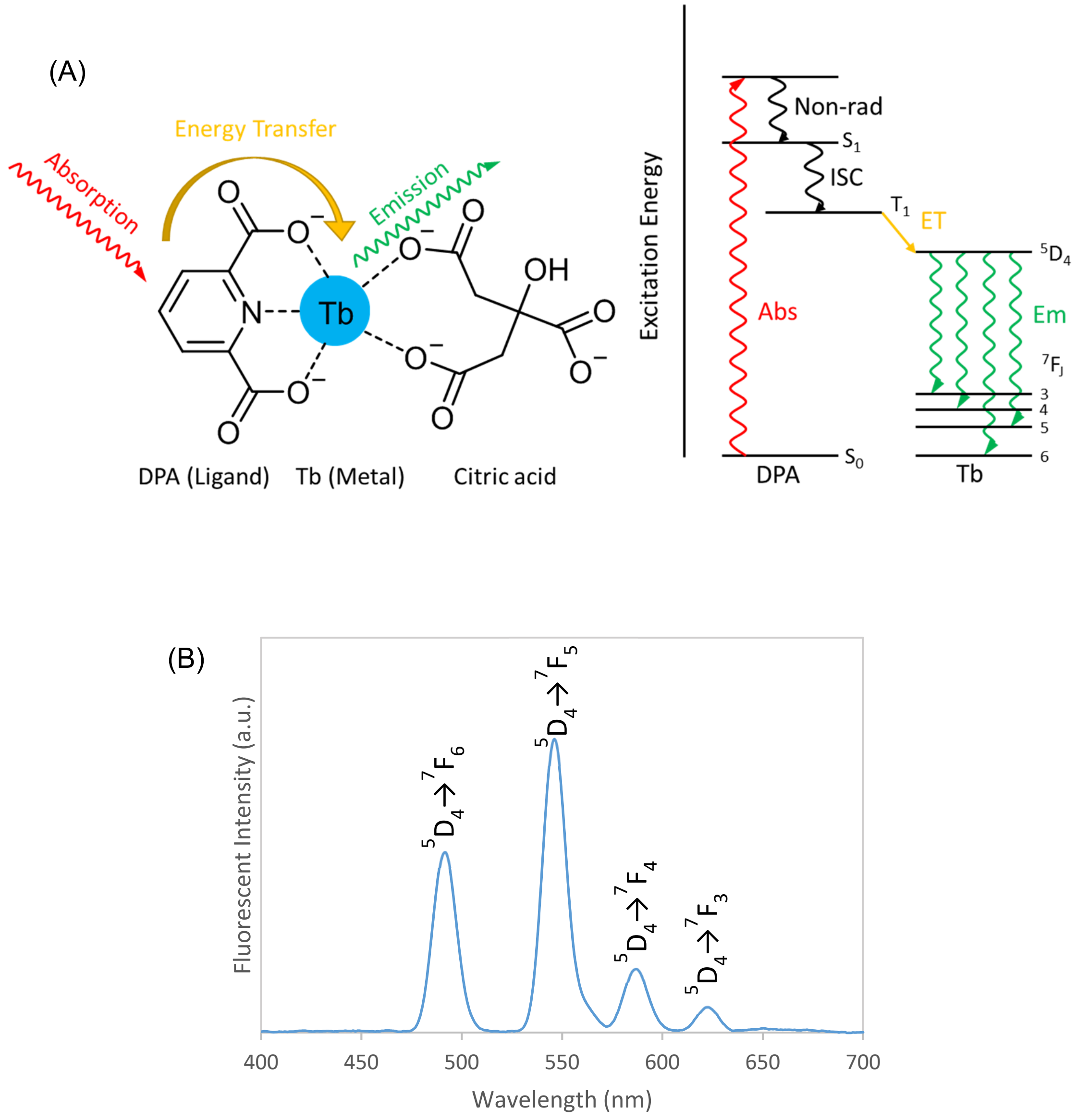

Figure S2. Fluorescence property of DPA-Tb complex
(A) Mechanism of charge transfer transition, (B) Fluorescence spectrum of DPA-Tb complex

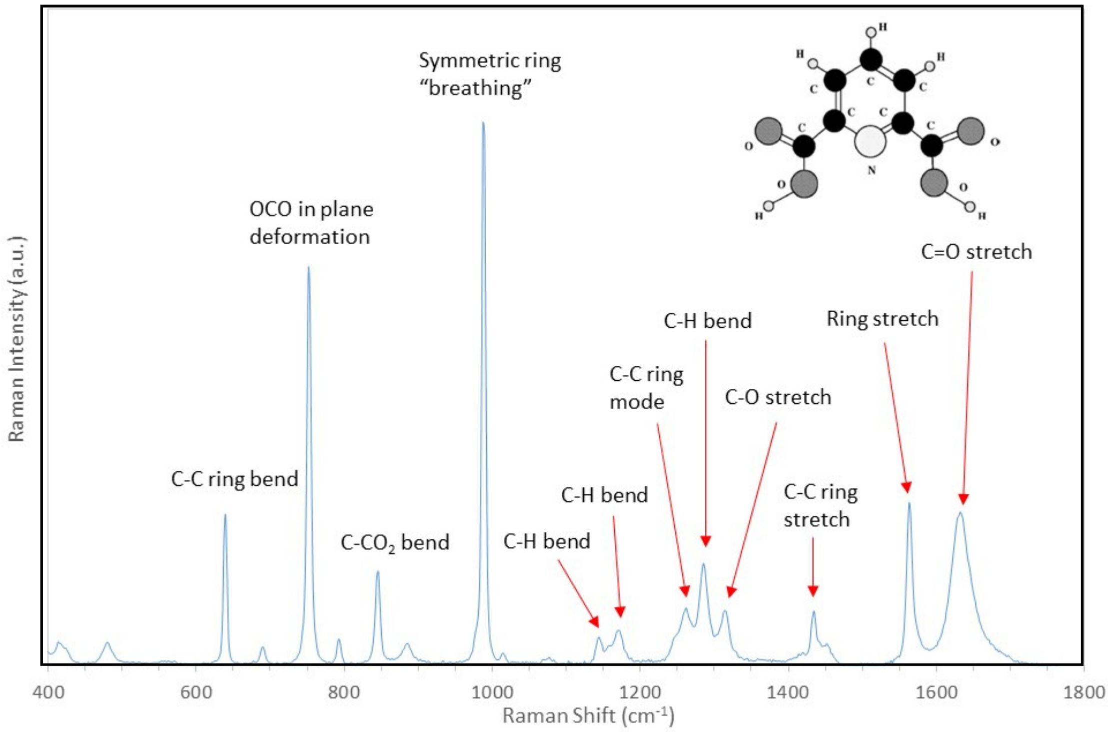

Figure S3 Raman spectrum of DPA.

RAMAN touch (Nano photon)

Excitation：533nm, Exposure time：10s

Cumulative Number:5, Laser power: 5mW

Table S1 Raman spectral bands of DPA powder and their attribution

Raman Shift (cm^{-1})	Assignment	Scale
**639**	C − C ring bend	Strong
**748**	OCO in-plane deformation	Strong
**796**	C − H out-of-plane	Very weak
**844**	C − CO_2 bend	Weak
**885**	C − H out-of-plane	Medium
**933**	C − H out-of-plane	Very weak
**987**	Symmetric ring “breathing”	Very strong
**1080**	Trigonal ring “breathing”	Very weak
**1146**	C − H bend	Weak
**1170**	C − H bend	Weak
**1262**	C − C ring mode	Medium
**1289**	C − H bend	Medium
**1317**	C − O stretch	Medium
**1437**	C − C ring stretch	Medium
**1569**	Ring stretch	Strong
**1638**	C = O stretch	Very strong
